# Clinical Audit of COPD Patients Requiring Hospital Admissions in Spain: AUDIPOC Study

**DOI:** 10.1371/journal.pone.0042156

**Published:** 2012-07-31

**Authors:** Francisco Pozo-Rodríguez, Jose Luis López-Campos, Carlos J. Álvarez-Martínez, Ady Castro-Acosta, Ramón Agüero, Javier Hueto, Jesús Hernández-Hernández, Manuel Barrón, Victor Abraira, Anabel Forte, Juan Miguel Sanchez Nieto, Encarnación Lopez-Gabaldón, Borja G. Cosío, Alvar Agustí, on behalf of the AUDIPOC Study Group

**Affiliations:** 1 Instituto de Investigación, Hospital 12 de Octubre, Madrid Spain; 2 Centre for Biomedical Research on Respiratory Diseases (CIBERES). Instituto de Salud Carlos III, Madrid, Spain; 3 Instituto de Biomedicina de Sevilla (IBiS), Hospital Universitario Virgen del Rocío, Seville, Spain; 4 Hospital Universitario Marqués de Valdecilla, Santander, Spain; 5 Complejo Hospitalario de Navarra, Pamplona, Spain; 6 Hospital Nuestra Sra. de Sonsoles, Ávila, Spain; 7 Complejo Hospitalario San Millán, Logroño, Spain; 8 Hospital Universitario Ramón y Cajal, IRYCIS, Madrid, Spain; 9 Centre for Biomedical Research on Epidemiology and Public Health (CIBERESP), Instituto de Salud Carlos III, Madrid, Spain; 10 Department of Statistics and Operational Research, Universidad de Castellón, Castellón, Spain; 11 Hospital Universitario Morales Meseguer, Murcia, Spain; 12 Hospital Virgen de la Salud, Toledo, Spain; 13 Hospital Universitario Son Espases, Balearic Island, Spain; 14 Instituto del Tórax, Hospital Clìnic, Barcelona, Spain; Public Health Agency of Barcelona, Spain

## Abstract

**Backgrounds:**

AUDIPOC is a nationwide clinical audit that describes the characteristics, interventions and outcomes of patients admitted to Spanish hospitals because of an exacerbation of chronic obstructive pulmonary disease (ECOPD), assessing the compliance of these parameters with current international guidelines. The present study describes hospital resources, hospital factors related to case recruitment variability, patients’ characteristics, and adherence to guidelines.

**Methodology/Principal Findings:**

An organisational database was completed by all participant hospitals recording resources and organisation. Over an 8-week period 11,564 consecutive ECOPD admissions to 129 Spanish hospitals covering 70% of the Spanish population were prospectively identified. At hospital discharge, 5,178 patients (45% of eligible) were finally included, and thus constituted the audited population. Audited patients were reassessed 90 days after admission for survival and readmission rates. A wide variability was observed in relation to most variables, hospital adherence to guidelines, and readmissions and death. Median inpatient mortality was 5% (across-hospital range 0–35%). Among discharged patients, 37% required readmission (0–62%) and 6.5% died (0–35%). The overall mortality rate was 11.6% (0–50%). Hospital size and complexity and aspects related to hospital COPD awareness were significantly associated with case recruitment. Clinical management most often complied with diagnosis and treatment recommendations but rarely (<50%) addressed guidance on healthy life-styles.

**Conclusions/Significance:**

The AUDIPOC study highlights the large across-hospital variability in resources and organization of hospitals, patient characteristics, process of care, and outcomes. The study also identifies resources and organizational characteristics associated with the admission of COPD cases, as well as aspects of daily clinical care amenable to improvement.

## Introduction

The existence of variations in clinical practice and clinical appropriateness has been recognized for decades [1]. Two methods, both developed in the late 1980s, of exploring and dealing with these variations are Evidence-Based Medicine and Clinical Audits [2], [3]. These approaches are of particular relevance for the development of evidence-based clinical practice guidelines and the generation of real-life information that may eventually feed-back to the further refinement of guidelines [4].

Chronic Obstructive Pulmonary Disease (COPD) is a major health problem of increasing incidence. COPD is currently the 5^th^ most common cause of death in the world, with the World Health Organization (WHO) predicting that it will rank 4^th^ by 2030 [5]. In Spain, the prevalence of COPD is about 10% of the adult population [6]. Many COPD patients suffer episodes of exacerbation (ECOPD) during the course of their disease which impact negatively on their health status and prognosis, and constitute a major portion of the total health care costs of the disease [7]-.

Two British multicentre COPD clinical audits reported wide variability in interventions and outcomes. [8], [9] Other smaller audits have followed [10], [11], showing wide variations between different hospitals and between different countries in patient care, which is frequently not consistent with published guidelines. Interestingly, considerable variation in case recruitment and characteristics of cases across hospitals has also been described. This variations have been traditionally associated to temporal and geographical factors [12 14]. However, no studies have been carried out to assess the importance of these variations and the factors associated to them. Identifying organizational or clinical factors potentially associated with the admission to hospital and diagnosis of COPD cases (“case recruitment”) may therefore be of interest in efforts to improve the quality of health care afforded to such patients.

This paper presents for the first time the results of the AUDIPOC study, a national clinical audit carried out in Spain that sought to: 1) describe hospital resources and organizational patterns of hospitals in Spain delivering care for patients with ECOPD 2) analyse the variability among hospitals in COPD case recruitment and associated factors; 3) describe patient characteristics, clinical interventions and outcomes, both at the patient and hospital level; and 4) inform on the adequacy of care as per current clinical practice guidelines.

## Methods

### Study Design and Ethics

The methods of the AUDIPOC study have been described in detail elsewhere [15]. Briefly, AUDIPOC is a cross-sectional study with prospective case ascertainment of consecutive ECOPD hospital admissions from November 1^st^ to December 31^st^, 2008, and retrospective data gathering from medical records. Patients were followed-up for 90 days after hospital admission with a view to include in the analysis two clinically relevant outcomes: mortality (in-hospital and out-hospital) and readmissions. The ethics committee of each participating hospital approved the study protocol. Due to the non-interventional nature of the study and the need of blindly evaluating the clinical performance, an informed consent was waived.

### Participating Hospitals

All 225 acute care hospitals of the public Spanish National Health System listed in the 2008 Registry of the Ministry of Health [16] were invited to participate. Each hospital’s catchment population was estimated from the proportion of the corresponding regional population census (January 1^st^, 2009) that was assigned for admission to that particular hospital [17].

### Ascertainment of Cases

The inclusion of patients in the AUDIPOC study followed a two-step process. First, clinical notes of all cases hospitalized by the Emergency Department (ED) were reviewed daily to identify one or more of 13 clinical conditions compatible with the diagnosis of ECOPD (table 1); these patients were labelled as interim ECOPD cases. Second, these cases were reassessed at hospital discharge against a list of definite inclusion and exclusion criteria (table 1) to identify cases with a clinical diagnosis of ECOPD, that were labelled as definite ECOPD cases [15].

**Table 1:** Provisional and definitive inclusion and exclusion criteria.

Provisional inclusion of the patient upon admission
1. CPOD or chronic pulmonary obstructive disease
2. COB or chronic obstructive bronchitis
3. CB or chronic bronchitis
4. CAO or chronic airflow obstruction
5. CAL o chronic airflow limitation
6. Obstructive lung disease
7. Asthmatic bronchitis with or without reference to acuteness, exacerbations, dyspnoea, bronchospasms, or respiratory insufficiency
8. Respiratory infection, excluding pneumonia
9. Bronchial infection
10. Chronic, acute, or exacerbated respiratory failure, not associated with a causal effect other than CPOD
11. Filial, non-filial, or undetermined dyspnoea
12. Non-specific or non-filial respiratory pathology under study
13. Heart Failure IF acute pulmonary oedema is not explicitly mentioned and IF accompanied by any of the terms previously described
**Inclusion and exclusion criteria**
**a. Definitive inclusion criteria (at least one)**
1. Admitted principally for eCPOD diagnosis
2. Admitted for “respiratory pathology” [respiratory infection without radiological infiltration or pleural effusion (OR) respiratory failure (OR) right heart failure(OR) bronchitis (OR) bronchospasms (AND) [historical diagnosis of CPOD (OR) a documented FEV1/FVC <0.70 in the absence of other obstructive diseases suchas asthma or bronchiolitis]
**b. Definitive Exclusion Criteria (any of the following):**
1. Specific diagnosis: pulmonary oedema, pneumonia, pulmonary embolism, pneumothorax, rib fractures, aspiration, pleural effusion, etc. upon admission
2. Other associated respiratory pathology that determines treatment: pulmonary fibrosis, kyphoscoliosis, obesity-hypoventilation, neuromuscular pathology,upper airway obstruction, bronchiectasis, extensive tuberculosis sequelae, asthma, bronchiolitis or uncontrolled brochogenic carcinoma
3. Pathology outside the lungs that determines treatment: major cardiopathy with chronic heart failure, evolved dementia, extended neoplasia, liver orkidney failure, or other situations at the discretion of the researcher

These criteria are evaluated on the discharge report and clinical history. The cases included are those that have at least one inclusion criteria and no exclusion criteria.

### Data Acquisition and Processing

Hospitalization and follow-up data were obtained from clinical records and entered into a web-based application that was monitored daily to identify errors, inconsistencies and missing values during the audit. Once the audit had ended, two quality controls were established. Firstly, independent auditors re-entered data for 28 relevant variables for a random sample of 1897 patients (15% of all interim cases). Secondly, after a preliminary data description that was made to identify extreme values and inconsistencies, the database entered a data cleaning process [18]. Those values considered extreme or found to have inconsistencies with other related variables were sent to local investigators to check and send back the correct value.

### Guidelines Adherence Evaluation

The main recommendations regarding hospital care of ECOPD patients were identified from three different guidelines (GOLD 2010 [19], NICE 2010 [20] and SEPAR/ALAT 2009 [21]), and the degree of compliance with these recommendations was investigated in the AUDIPOC database.

### Statistical Analysis

Results at a patient level are presented as percentages or medians, interquartile ranges (IQR) and ranges, as appropriate. Results at a hospital level are presented as medians IQR and ranges of data grouped for all patients within each hospital (i.e. clustered). Inter-rater agreement between the initial set of data and the re-entered by independent auditors was calculated as Cohen’s kappa coefficient. Between-hospital variability in case recruitment was modelled using Bayesian multivariate analysis [22], [23]. Bayesian analysis is a method of statistical inference that allows the investigator to explicitly incorporate the distribution of prior beliefs and expert knowledge (prior probability distribution) concerning parameters such as means, variances and regression coefficients underlying random variables, with the currently observed data and the assumed probability model to obtain posterior probability distributions. To determine which hospital resource and organizational related attributes were associated with the variable “ratio of observed to expected number of interim cases,” we used a Poisson probability regression model. For the “proportion of interim cases ultimately considered definite cases,” a binomial probability regression model was fitted.

The above models were built allowing for the quantification of the extra-variability in the response variables via a random effects term. Uninformative prior distributions were used to assign prior probabilities to all values for each parameter, including the regression coefficients and the variance associated with the hospital-level random effects term. The Markov Chain Monte Carlo (MCMC) method was used [24], to simulate posterior distributions of all parameters in the final model. In order to maximize the quality of the sampling of the posterior distributions, one million iterations were run for each of the two models; one half to verify convergence, and the other half for statistical inference. Results were expressed with the mean posterior probability and its 25–75% limits of credibility, and with an estimated average Odds Ratio. These credible intervals indicate that the true population parameter lies in this interval with a probability of 50%.

## Results

A total of 11,564 interim ECOPD cases were hospitalized during the study period. At discharge, 5,178 patients fulfilled all the inclusion and none of the exclusion criteria and were therefore included in the audit as definite ECOPD cases. Not-included cases were slightly older (78 vs. 75 yrs.), more often females (47 vs. 12%) and were less frequently diagnosed on admission with conditions related to COPD (20 vs. 80%) or at discharge with a primary diagnosis of COPD (18 vs. 82%). Regarding the internal consistency of the data recorded, it is of note that the inter-rater agreement was high, with 68% of Cohen’s kappa coefficients >0.61 and only one <0.40 (Table S1 in the online Appendix).

### Characteristics of Participating Hospitals

A total of 129 hospitals (57% of all those potentially eligible) from all 17 Spanish regions participated in the AUDIPOC study. The proportion of participating hospitals and the regional population coverage varied across regions (Table 2). We estimated that the AUDIPOC study covered a total population of 31,744,972, representing about 70% of the Spanish population.

**Table 2:** Participating hospitals and population coverage by region.

Regions	Eligible hospitals	Participatinghospitals	Population assigned for admission	Total population	Population coveredby the study
Andalusia	31	17 (55%)	4882253	8150467	60%
Aragon	10	2 (20%)	666761	1313735	51%
Asturias	9	5 (56%)	942376	1058923	89%
Balearic Islands	8	6 (75%)	1025658	1070066	96%
Basque Country	11	11 (100%)	2045163	2136061	96%
Canary Islands	8	4 (50%)	1432866	2076585	69%
Cantabria	4	3 (75%)	560406	576418	97%
Castile-La Mancha	15	7 (47%)	1546687	2022647	76%
Castile and León	15	13 (87%)	2500503	2510545	99%
Catalonia	27	10 (37%)	3006371	7290292	41%
Extremadura	10	3 (30%)	630481	1080439	58%
Galicia	15	9 (60%)	2118741	2738930	77%
La Rioja	2	2 (100%)	307676	315718	97%
Madrid	20	15 (75%)	5317786	6295011	84%
Murcia	10	4 (40%)	833349	1433383	58%
Navarre	4	4 (100%)	563900	596236	95%
Valencia	26	14 (54%)	3363995	4991789	67%
TOTAL	225	129 (57%)	31744972	45657245	70%

Table 3 shows the inter-hospital variation regarding hospital resources and organization of care. The size of the catchment population, the number of hospital beds and the total number of hospital admissions varied widely (about 20-fold) between participating hospitals. These inter-hospital differences increased further (60-fold) for the number of ECOPD admissions in the year prior to the audit, total number of faculty physicians (50-fold) and total number of pulmonologists in the faculty (30- fold). There was a 10-fold variation between hospitals in relation to the number of internists.

**Table 3:** Characteristics of participating hospitals (N = 129).

Variable	Reported	%	Median	Q1–Q3	Min.– Max.
Catchment population (hab)	129		224076	136036–340458	44000–787000
Total beds per hospital	129		373	192–599	61–1352
Patients admitted by hospital in 2007	129		14573	7246–21165	600–90000
COPD patients admitted per hospital in 2007	129		377	199–591	56–3500
Total staff physicians within the hospital	129		293	151–519	25–1417
Internal Medicine Staff members	129		12	8.00–18	5–50
Pulmonary Medicine Staff members	109		8	5.00–12	1–30
Hospital case-mix index 2007	129		1.3	1.0–1.6	0.7–2.2
Hospitals with residents in training	129	79			
University Hospital	129	50			
Pulmonology Unit	129	84			
Yes, with hospital ward		61			
Yes, without hospital ward		23			
No		16			
Lung function laboratory available	129	83			
Availability of non-invasive ventilation	129	95			
Intensive care/High Dependency Unit	129	90			
Admissions ward	129	87			
Pulmonary physicians on duty on site	79	61			
Written protocol for COPD	129	44			
Formal pulmonary rehabilitation programme	129	28			
Availability for transferring COPD cases to another hospitals	129	43			
Early discharge scheme/hospital at home	129	20			
Triage by physicians	129	40			
Access to electronic/digital information	129	78			
Number of interim ECOPD cases recruited	129		80	41–136	8–365
Number of definite ECOPD cases recruited	129		37	25–60	8–134

Q1–Q3: interquartile range.

### Analysis of Hospital Variability in Case Recruitment

Table 4 displays the mean and 50% credible intervals of the posterior distribution of regression coefficients in the final models, as well as the corresponding average odds ratio (OR). The positive/negative signs in front of their values indicate the same/opposite direction of effects. The number of interim ECOPD cases recruited was positively associated with variables somehow related to COPD awareness such as number of cases admitted in the year prior to the study, existence of a COPD clinical management protocol, or the availability of respiratory physicians in the ED. By contrast they were negatively associated with variables related to hospital size and complexity, including case-mix index, number of beds, existence of an early discharge scheme or domiciliary hospitalisation, number of respiratory physicians, or being a university-affiliated hospital (Table 4, upper panel). On the other hand, the proportion of interim cases that became definite was positively associated with hospital size and complexity and negatively associated with hospital COPD awareness (exceptions were university affiliated and medical training hospitals, for which the probability of an interim case becoming definite decreased) (Table 4, lower panel). Interestingly, both the number of interim ECOPD cases and the proportion of definite cases rose with the availability of hospital documents in electronic format. In any case, a large component of the hospital-related variance in the number of interim ECOPD cases recruited initially (calculated as a 45-fold change) and the proportion of interim cases that become definite (calculated as a 17-fold change) remained unexplained by the models fitted (Table 4).

**Table 4:** Multivariate Bayesian analysis showing posterior distributions for the regression coefficients associated with recruitment hospital performance.

	Mean posterior probability	25% Limit of credibility	75% Limit of credibility	OR
Response variable: ratio of interim recruited to expected COPD cases				
Intercept	−2.50	−3.18	−1.79	
Total patients admitted in 2007 (log N)	0.29	0.19	0.38	1.33
Access to electronic/digital information (Yes)	0.23	0.13	0.34	1.26
COPD patients admitted in 2007 (log N)	0.20	0.12	0.28	1.23
Access to pulmonologist in the ED (Yes)	0.13	0.03	0.25	1.15
Written protocol for COPD (Yes)	0.09	−0.01	0.19	1.09
Early discharge scheme/day hospital/hospital at home (Yes)	−0.31	−0.42	−0.21	0.73
Total hospital beds (log N)	−0.17	−0.28	−0.03	0.85
Hospital case-mix index (units)	−0.14	−0.26	0.00	0.87
University hospital (Yes)	−0.13	−0.25	−0.01	0.88
Pulmonary medicine staff (N)	−0.04	−0.06	−0.02	0.96
Hospital random effects (standard deviation)	0.70	−0.67	0.73	
Response variable: proportion of interim COPD cases that become definite				
Intercept	1.71	0.97	2.45	
Hospital Case-mix (units)	0.24	−0.00	0.49	1.27
Lung function laboratory (Yes)	0.40	0.15	0.65	1.49
Access to electronic/digital information (Yes)	0.34	0.15	0.54	1.40
Pulmonary physicians on duty on site (Yes)	0.18	−0.06	0.41	1.20
Pulmonary medicine staff (N)	0.03	0.00	0.05	1.03
University Hospital (Yes)	−0.72	−0.92	−0.52	0.49
Residents in training (Yes)	−0.34	−0.54	−0.14	0.71
Written protocol for COPD (Yes)	−0.31	−0.48	−0.12	0.74
COPD patients admitted for COPD in 2007 (log N)	−0.26	−0.38	−0.12	0.77
Non-invasive ventilation (Yes)	−0.26	−0.53	−0.00	0.77
Hospital random effects (standard deviation)	1.29	1.23	1.36	

OR: Odds Ratio.

### Characteristics of Audited Patients

Table 5 presents the main clinical characteristics, interventions and outcomes of the 5,178 patients included in the audit, at different time points and at both the patient and hospital levels. Additional information can be found in the online supplement (Table S2 in the online Appendix). There was large variability in patient characteristics, interventions and outcomes across hospitals. Gender, age, smoking status, comorbidity, general health status (e.g. performance status), anaemia, peripheral oedema, serum albumin and creatinine levels and the frequency and severity of respiratory failure (Table S2 in the online Appendix) varied widely between patients treated in different hospitals. Further differences between hospitals were observed in relation to the availability of spirometric data, arterial blood gas analysis at the ED, and prescription of oxygen therapy, ventilation support, antibiotics, systemic/inhaled steroids, long-acting ß_2_ agonists (LABA) and long-acting muscarinic antagonists (LAMA), as well as for other treatments (Table 5). In-hospital mortality ranged from 0 to 35% (median  = 4.5%, IQR  = 1.3–7.7%). Length of stay (LOS) ranged from 4 to 65 days (median  = 8 days, IQR  = 7–10 days) and all-cause hospital readmissions from 0 to 62% (median 34%, IQR 28–42%). Mortality during follow-up ranged from 0 to 38% (median 6%, IQR 2–9%) and the overall mortality across hospitals ranged from 0 to 50% (median 12%, IQR 8–15%).

**Table 5:** Selected patient characteristics, clinical interventions and outcomes. Estimation at patient level and at hospital level.

Variables	At patient level (N = 5.178)		At hospital level (N = 129)		
	N	% or median (IQ limits)	Group data median	IQ limits	Range limits
Before admission					
Gender (men)	5178	87	90	82–94	46–100
Age (years)	5178	75(68–80)	75	73–77	63–85
Current smoker(yes)	4500	30	29	22–38	0–64
Comorbidity >1 (yes)	5178	38	38	26–46	9–88
Performance status (moderate to severe limitations)	3485	51	37	18–53	0–89
Documented spirometry (yes)	4191	73	63	42–76	0–100
Oxygen therapy (yes)	3403	39	25	17–34	0–75
Non-invasive ventilatory support (yes)	3403	5.2	6	3–9	0.8–22
Previous admissions with ECOPD (yes)	5178	74	74	65–81	41–100
On admission					
Arterial blood gases (yes)	5178	90	95	88–100	33–100
pH (units)	4630	7.41(7.37–7.44)	7.4	7.39–7.42	7.25–7.45
PaCO_2_ (mmHg)	4628	45(38–55)	46	43–49	38–76
PaO_2_, (mmHg)	4627	57(49–66)	56	53–60	30–69
Chest x ray (yes)	5178	98	100	97–100	27–100
EKG (yes)	5178	85	90	79–97	16–100
During hospitalization					
Admitted under Respiratory physician (yes)	5178	53	56	26–74	0–100
Acidosis (pH<7.35) at any time (yes)	5178	19	17	12–27	0–67
Admitted to ICU/HDU (yes)	5178	2.4	0	0–4	0–25
Short Acting Beta Adrenergics (yes)	5178	88	93	85–97	11–100
Short Acting Anti Cholinergics (yes)	5178	89	94	88–100	15–100
Inhaled steroids (yes)	5178	40	42	17–62	0–100
Systemic steroids (yes)	5178	92	94	89–98	50–100
Antibiotics (yes)	5178	90	92	86–95	55–100
Oxygen therapy (yes)	5178	96	98	95–100	53–100
Ventilatory support (yes)	5178	11	11	4–18	0–67
Death in hospital (yes)	5178	5	4.5	1.3–7.7	0.0–35.3
Length of Stay (days)	5178	8(6–12)	8	7–10	4–65
At discharge					
Long Acting Beta Adrenergics	4919	82	80	70–87	43–100
Long Acting Anti Cholinergics	4919	67	67	58–77	25–100
Inhaled steroids	4919	84	81	74–89	56–100
Systemic corticosteroids	4919	74	73	62–81	13–100
Antibiotics	4919	53	49	34–67	8–100
Oxygen therapy	4919	45	43	33–53	7–92
Non-invasive ventilatory support	4919	6	5	0–9	0–25
90 days follow up since admission					
Readmissions from all causes	4919	37	34	28–42	0–62
Readmissions from COPD	4919	28	26	18–33	0–54
Death at follow up	4919	6.9	6	2–9	0–38

N: Number of cases that reported data. COPD: Chronic Obstructive Pulmonary Disease. IQ limits: interquartile limits. Range limits: total range limits.

### Compliance with Clinical Practice Guidelines

Compliance with clinical practice guidelines is summarised in Table 6 (with further information available in Table S3 in the online Appendix). Although considerable variability at the hospital level was also observed, compliance with recommendations regarding diagnosis or in- hospital treatment revealed high standards of care. In contrast, the level of information included in the final discharge report was not of a high standard, since recommendations related to general health practices and life-style improvements were given in written form to less than 50% of discharged patients (Table 6).

**Table 6:** Guideline statements related to clinical findings GOLD (2010)/NICE (2009)/SEPAR-ALAT (2009).

Summary Statements	AUDIPOC results for patients grouped by hospital			
Clinical findings	Variable	Median	IRQ	Min-Max
An exacerbation of COPD is characterised by a change in the patient’sbaseline dyspnoea, cough, and/or sputum production or colour	Increased dyspnoea	96	93–100	84–100
	Increased sputum	64	54–72	9–100
	Increased purulence	88	80–100	17–100
	None of the symptoms	0	0–4	0–13
	Anthonisen Type I	40	30–49	7–100
	Anthonisen Type II	26	19–32	0–50
	Anthonisen Type III	31	23–40	0–91
**Diagnosis**	**Variable**	**Median**	**IRQ**	**Min-Max**
For patients that require hospitalisation, measurement of arterial bloodgases is important to assess the severity of an exacerbation.	Cases with a blood gas analysisin the emergency room	95	88–100	33–100
	Inspired oxygen concentrationrecorded in the ED	93	73–100	0–100
**Oxygen therapy**	**Variable**	**Median**	**IRQ**	**Min-Max**
Oxygen therapy is the cornerstone of hospital treatment of COPDexacerbations and Supplemental oxygen should be titrated to improve thepatient’s hypoxemia	Cases receiving oxygen duringadmission	98	95–100	53–100
	Pulse-oxymetry while receivingoxygen- therapy	98	86–100	0–100
**Bronchodilators**	**Variable**	**Median**	**IRQ**	**Min-Max**
Management of COPD exacerbations involves increasing the doseand/or frequency of existing short-acting bronchodilator therapy, preferablywith a ß_2_ agonist.	Cases on short-actingbronchodilators	98	94–100	61–100
	Cases on short-acting ß2 agonists	93	85–97	11–100
	Cases on ipratropium	94	88–100	15–100
**Antibiotics**	**Variable**	**Median**	**IRQ**	**Min-Max**
Antibiotics should be given to patients with three cardinal symptoms,with two cardinal symptoms if purulence of sputum is one of the twosymptoms, and patients that require mechanical ventilation	Cases on antibiotics withthree cardinal symptoms	98	90–100	50–100
	Cases on antibiotics with anincrease in sputum purulence	97	91–100	0–100
	Cases on ventilatorsupport receiving antibiotics	100	83–100	0–100
**Steroids**	**Variable**	**Median**	**IRQ**	**Min-Max**
In the absence of significant contraindications oral corticosteroids shouldbe used, in conjunction with other therapies, in all patients admitted to hospitalwith an exacerbation of COPD.	Cases on oral or intravenousglucocorticosteroids	94	89–98	50–100
**Discharge report**	**Variable**	**Median**	**IRQ**	**Min-Max**
Opportunities for prevention of future exacerbations should be reviewedbefore discharge, with particular attention to smoking cessation, currentvaccination (influenza, pneumococcal vaccines), knowledge of current therapyincluding inhaler technique and how to recognize symptoms of exacerbations.	Anti-tobacco instructions inactive smokers	43	23–63	0–100
	Influenza vaccination instructions	0	0–7	0–100
	Pneumococcal vaccinationinstructions	0	0–3	0–100
	Nutritional instructions	37	21–53	0–100
	Inhaler technique instructions	7	0–19	02100
	Programmed visit after discharge	95	89–100	30–100

## Discussion

This is the first national clinical audit of patients hospitalized in Spain because of ECOPD. Given the high percentage of population coverage the results should provide an accurate description of the clinical characteristics of ECOPD cases, current clinical practice models, and outcomes of ECOPD treatment in Spain. Further, our results identify the hospital characteristics associated with the admission of ECOPD patients and confirm, for the Spanish National Health System, previous findings on different health-care systems concerning the variability of available resources, clinical presentation and outcomes of patients admitted to these hospitals. Finally, our study provides novel information relating to the degree of actual compliance with international guidelines. Taken together, a proactive approach to the management of this information should contribute to improvements in organizational aspects of care given to COPD patients.

### Previous Studies

Most previous ECOPD audits included small samples of patients or hospitals, or focused on particular aspects of clinical care. Further to this, those studies involving large numbers of patients were mainly based on administrative databases [25]–[27]. To our knowledge, only two other nationwide clinical audits of patients hospitalized with ECOPD, and which involved the prospective recruitment of cases and collection of patient clinical record-based data, have been published to date. Both of these studies were carried out in the United Kingdom. The first was performed in 2003 and included 234 participating hospitals and 7514 patients. Median inpatient mortality was 7% (between-hospital IQR 3%–11%), total mortality was 15% (IQR 9%–21%), median LOS was 6 days (IQR 3–11 days) and the re-hospitalization rate was 31% (IQR 22%–40%) [8]. The second study, undertaken in 2008 and which included 232 participating hospitals and 9716 patients, reported similar updated results [9]. Overall, the results of these two studies are in keeping with those of AUDIPOC. Minor differences may be related to the distinctiveness of the British and Spanish health systems as well as to differences in the inclusion criteria used in the respective studies.

### Interpretation of Findings

The general clinical profile of the patients included in the AUDIPOC study corresponded mostly to that of elderly persons (a third of whom were still smokers) with a history of previous ECOPD hospitalizations and frequent comorbidities (mostly cardiovascular). At the ED they complained of increased dyspnoea with purulent sputum. By and large, treatment during hospitalization and at discharge followed international recommendations (Tables 6 and S3). There were, however, relatively few documented interventions aimed at promoting smoking cessation, an active life-style (including rehabilitation prescription) and/or influenza or pneumococcal vaccination. Importantly, re-hospitalizations were frequent and there was remarkably high all-cause mortality (11.6%).

A more detailed analysis of our results, however, showed that there were marked variations across hospitals in terms of patient characteristics, process of care, adherence to guidelines (Tables 6 and S3), and outcomes. Although part of this variability can be explained by the relatively small number of cases provided by some hospitals, it is more likely due to one or more of the following: (1) heterogeneity of the participating hospitals in terms of size, resources and organization, case recruitment, complexity of health care delivery, (2) heterogeneity of the disease itself [28] as well as diversity of interventions undertaken; (3) thoroughness and accuracy of clinical record data collection; or (4) other, still unidentified, factors not included in the analysis, such as those related to geographical location [14]. In order to gain further insight into the relative contribution of each of these, we first investigated what hospital resources and organizational variables could be identified to explain the recruitment of ECOPD cases into the audit. To this end, we used a Bayesian approach because of its flexibility to study complex models and databases, and the fact that it generates posterior probability distributions that facilitate the interpretation of regression coefficients [29]. This identified a number of explanatory variables that seem to act in opposing directions with respect to the number of interim COPD cases and the proportion of definite cases (Table 4). Hospital size and complexity attributes were associated with admitting fewer interim cases and selecting more definite cases from these (i.e. a more refined selection strategy), whilst the COPD awareness dimension facilitates the admission of more interim cases and selection of fewer definite cases (i.e. a less refined selection strategy). The association of access to electronic/digital information with the number of interim and definite cases suggests that the use of information technologies may increase the identification of cases and, possibly, improve the audit process. In any case, a large component of hospital-related variance remained unexplained, suggesting that the clinical profile of patients included in the study also varied markedly across hospitals. Differences in reported outcomes could also be due to discretionary patterns in the process of care itself. To this extent, an exhaustive study of these variations might offer the best chance for resolving potential differences in quality of treatment provided to ECOPD patients.

Finally, our study provides novel information on the degree of real-life compliance with international guidelines which, overall, was acceptable. The study identified, however, that general recommendations concerning a healthy life-style, such as detailed instructions on how to stop smoking, how to improve nutrition or undertake more daily physical activity, or the provision of information concerning the advantages of influenza and pneumococcal vaccination, were often not provided. This issue has been recently audited and specific national recommendations have been issued [30], [31].

### Strengths and Limitations

The application of strict inclusion/exclusion criteria has likely resulted in the inclusion in the AUDIPOC study of a relatively “pure” ECOPD cohort, with few patients incorrectly included. As such, the study’s results can be generalized to all patients admitted with ECOPD. An additional strength of this study is that the auditors were reasonably consistent in their re-entering of data, thus supporting the quality of the data retrieving and entry process (Table S1). On the other hand, however, a potential limitation of this study, which is intrinsic to any clinical audit, is that medical charts were used as the data source, so some missing and inconsistent values were unavoidable. To address this limitation, a thorough process of database screening and editing was undertaken and the number of extreme or inconsistent values was notably reduced. However, missing data values still remained that tended to cluster in related variables, thus contributing to cross-correlation among them and rendering the multivariate analysis particularly challenging.

### Conclusions

The AUDIPOC study is the first national audit on patients hospitalized in Spain because of ECOPD. Our results confirm previous studies from other countries and show significant variability in terms of the resources and organization of hospitals, process of care and outcomes. The study also identifies for the first time a number of resources and organizational characteristics of hospitals that may influence the routine admission of COPD cases for hospitalization, pinpointing therefore to several improvable organizational aspects. The issue of compliance with clinical practice guidelines in real life was also addressed, with some aspects that are amenable to improvement of daily clinical care also identified.

## Supporting Information

Table S1 **Consistency analysis of variables included in the study.**
(DOCX)

Table S2 **Additional patient characteristics, clinical interventions and outcomes. Estimations at patient level and at hospital level.**
(DOCX)

Table S3 **Additional guidelines assessment.**
(DOCX)
