# Supplementary material for: Clinical Audit of COPD Patients Requiring Hospital Admissions in Spain: AUDIPOC Study
Source: PLoS One. 2012 Jul 31;7(7):e42156. doi: 10.1371/journal.pone.0042156 (PMC3418048; doi:10.1371/journal.pone.0042156)
Supplement: Table S1 — Consistency analysis of variables included in the study. (DOCX) [file pone.0042156.s001.docx]

Table S1. Consistency analysis of variables included in the study.

| Variable | N | Prevalence | Agreement | Expected agreement | Kappa (95%CI) |
| --- | --- | --- | --- | --- | --- |
| Decision for inclusion in AUDIPOC study | 1012 | 0.47 | 0.96 | 0.50 | 0.56 (0.47-0.65) |
| IC: COPD as primary diagnostic | 523 | 0.82 | 0.86 | 0.71 | 0.81 (0.72-0.89) |
| IC: COPD as secondary diagnostic | 453 | 0.27 | 0.80 | 0.59 | 0.66 (0.57-0.74) |
| EC: Other respiratory pathology that determines | 523 | 0.2 | 0.82 | 0.64 | 0.93 (0.87-0.99) |
| EC: Other airway pathology that determines care | 523 | 0.18 | 0.85 | 0.71 | 0.54 (0.45-0.62) |
| EC: Heart failure | 523 | 0.23 | 0.90 | 0.64 | 0.50 (0.41-0.58) |
| EC: Thoracic neoplasm | 523 | 0.02 | 0.99 | 0.94 | 0.51 (0.42-0.59) |
| EC: Extra-pulmonary pathology that determines | 523 | 0.21 | 0.89 | 0.67 | 0.73 (0.64-0.81) |
| Number of data sources used | 453 | 0.02 | 0.46 | 0.20 | 0.78 (0.70-0.86) |
| Admission to ICU/HDU | 452 | 0.02 | 0.99 | 0.96 | 0.52 (0.43-0.61) |
| Comorbidity clinically relevant | 452 | 0.48 | 0.77 | 0.50 | 0.82 (0.73-0.91) |
| Change of sputum colour | 452 | 0.46 | 0.82 | 0.40 | 0.53 (0.44-0.62) |
| Peripheral oedema | 452 | 0.03 | 0.90 | 0.53 | 0.69 (0.62-0.76) |
| Treatment during 7 days previous to admission | 452 | 0.52 | 0.70 | 0.44 | 0.65 (0.59-0.72) |
| Spirometry previous to admission | 451 | 0.31 | 0.82 | 0.47 | 0.81 (0.69-0.93) |
| Availability of full spirometric data | 256 | 0.95 | 0.98 | 0.92 | 0.74 (0.65-0.84) |
| Blood gases at admission | 452 | 0.93 | 0.97 | 0.87 | 0.54(0.45-0.63) |
| Chest X-ray at admission | 451 | 0.97 | 0.98 | 0.84 | 0.47 (0.41-0.54) |
| Sputum microscopy at admission | 451 | 0.2 | 0.98 | 0.67 | 0.77 (0.68-0.86) |
| Systemic steroids during admission | 451 | 0.08 | 0.93 | 0.84 | 0.62(0.38-0.86) |
| Acidosis at any time during admission | 414 | 0.17 | 0.92 | 9.67 | 0.83 (0.74-0.92) |
| Arterial blood gases following acidosis | 61 | 0.8 | 0.85 | 0.61 | 1.00 (0.67-1.00) |
| Ventilatory support during admission | 441 | 0.1 | 0.97 | 0.81 | 0.72 (0.63-0.81) |
| Anti-tobacco instructions | 452 | 0.1 | 0.95 | 0.81 | 0.88 (0.79-0.97) |
| Influenza vaccination instructions | 452 | 0.09 | 0.80 | 0.69 | 0.65 (0.55-0.75) |
| In-hospital death | 452 | 0.05 | 0.99 | 0.91 | 0.79 (0.70-0.89) |
| Death on follow-up | 427 | 0.07 | 0.95 | 0.85 | 0.65 (0.55-0.75) |
| Readmissions during 90 days follow-up | 421 | 0.34 | 0.90 | 0.52 | 0.33 (0.28-0.37) |

IC: inclusion criteria. EC: exclusion criteria. ICU: intensive care unit. HDU: high-dependency unit.
