# Supplementary material for: Clinical Audit of COPD Patients Requiring Hospital Admissions in Spain: AUDIPOC Study
Source: PLoS One. 2012 Jul 31;7(7):e42156. doi: 10.1371/journal.pone.0042156 (PMC3418048; doi:10.1371/journal.pone.0042156)
Supplement: Table S2 — Additional patient characteristics, clinical interventions and outcomes. Estimations at patient level and at hospital level. (DOCX) [file pone.0042156.s002.docx]

Table S2. Additional patient characteristics, clinical interventions and outcomes. Estimations at patient level and at hospital level.

| Variables | At patient level  N=5.178 | | At hospital level  N=129 | | |
| --- | --- | --- | --- | --- | --- |
|  | N | %/Median  (IQ limits) | Group data median | IQ  limits | Range limits |
| Before admission | | | | | |
| Smoking status  Current smoker  Ex-smoker  Lifelong non-smoker | 4500 | 30  65  5 | 29  65  2.1 | 22-38  56-73  0-7 | 0-64  0-86  0-38 |
| Comorbidity  Cardiovascular disease  Heart failure  Diabetes Mellitus  Solid tumour | 5178 | 31  22  26  13 | 30  20  25  12 | 23-38  11-29  19-33  7-17 | 0-87  0-54  0-63  0-33 |
| Performance Status  Mild limitation (0-2)  Severe-very severe limitation (3-4) | 3485 | 49  51 | 30  37 | 17-44  18-53 | 0-92  0-89 |
| Body Mass Index  ≤29  >29 | 1931 | 63  37 | 56  32 | 0-70  0-46 | 0-100  0-100 |
| Social circumstances  Lives in own home  Residential or nursing home | 3376 | 91  9 | 92  8 | 87-100  0-13 | 0-100  0-67 |
| Spirometry previous to admission  No obstruction (FEV1/FVC>70)  Mild-moderate (Gold I-II)  Severe-very severe (Gold III-IV)  FVC, litres  FVC %  FEV1, litres  FEV1 %  FEV1/FVC % | 4191  2552  2552  2552  2474  2593  2533  2738  2579 | 73  10  27  63  2.2(1.7-2.7)  64(51-77)  1.1(0.8-1.4)  42(32-55)  51(42-62) | 63  5  20  53  0.09  90  0.09  96  91 | 42-76  0-12  8-30  37-65  0.06-0.1  70-100  0.07-0.1  84-100  69-100 | 0-100  0-56  0-100  0-100  0-0.1  0-100  0-0.1  0-100  0-100 |
| At admission | | | | | |
| COPD as principal diagnosis | 5178 | 81 | 86 | 74-94 | 24-100 |
| Treatment in seven days before admission | 4491 | 76 | 85 | 66-93 | 5-100 |
| Anthonisen criteria  Type I  Type II  Type III | 5178 | 43  27  30 | 42  26  31 | 33-50  19-32  23-40 | 7-100  0-50  0-91 |
| Heart rate | 4366 | 94(81-105) | 94 | 90-100 | 73-112 |
| Respiratory rate | 2452 | 24(20-28) | 24 | 20-28 | 14-35 |
| Blood pressure  High (SBP≥140 or DBP≥90)  Low (SBP≤90) | 4585 | 48  2 | 50  0 | 43-56  0-3 | 0-100  0-17 |
| Pedal oedema, % | 5178 | 26 | 26 | 19-33 | 0-53 |
| Arterial blood gases taken  Ratio PaO2/FiO2  Bicarbonate | 4627  4628 | 257(219-295)  28(25-31) | 252  28 | 242-270  27-29 | 143-329  21-37 |
| Ratio PaO2/FiO2  >300  200-300  <200 | 4627 | 24  61  16 | 20  61  15 | 13-32  53-69  9-22 | 0-63  0-92  0-100 |
| Microscopy or sputum culture % | 5178 | 26 | 17 | 8-40 | 0-85 |
| During admission | | | | | |
| Audit involvement of the patient’s physician on charge | 5178 | 15 | 13 | 2-27 | 0-100 |
| Oxygen therapy  Control pulse-oximetry | 5178 | 96  84 | 93 | 73-100 | 0-100 |
| Acidosis (pH< 7.35) at any time during admission  Second set of arterial blood gases  Oxygen after a pH<7.35 | 5178  781  529 | 19  68  90 | 17  50  100 | 12-27  30-75  92-100 | 0-67  0-100  0-100 |
| Ventilatory support  Non-invasive | 5178  582 | 11  93 | 11  9 | 4-18  3-17 | 0-67  0-56 |
| Serum albumin levels (g/l)  ≤3.5  >3.5 | 1669 | 3.7(3.3-4.0)  39  61 | 26  8  11 | 1-61  0-21  0-36 | 0-100  0-95  0-76 |
| Blood creatinine level (mmol/l)  ≤1.20  >1.20 | 4677 | 1.05(0.8-1.2)  78  22 | 95  79  21 | 88-100  71-87  13-29 | 33-100  50-100  0-50 |
| Anaemia (Hb<13.2 g/dl male, <12.2 female) | 4681 | 31 | 30 | 22-39 | 0-64 |
| Leucocytosis (>11.000) | 4680 | 50 | 50 | 40-57 | 14-100 |
| Long-acting ß2 agonist | 5178 | 34 | 29 | 11-56 | 0-100 |
| Long-acting muscarinic antagonist | 5178 | 20 | 10 | 3-29 | 0-79 |
| Methylxanthines | 5178 | 11 | 9 | 3-20 | 0-75 |
| Antioxidants | 5178 | 31 | 26 | 13-47 | 0-84 |
| At discharge | | | | | |
| Discharge to own home | 4919 | 93 | 88 | 82-92 | 25-100 |
| Short-acting ß2 agonist | 4919 | 51 | 43 | 30- 65 | 0-100 |
| Short-acting muscarinic antagonist | 4919 | 24 | 23 | 12-34 | 0-67 |
| Methylxanthines | 4919 | 11 | 9 | 3-18 | 0-50 |
| Antioxidants | 4919 | 27 | 20 | 10-36 | 0-75 |
| Discharge report with information on:  Inhaler technique instructions  Anti-tobacco instructions in active smokers  Nutritional instructions  Programmed visit after discharge  Influenza vaccination instruction  Pneumococcal vaccination instructions Pulmonary rehabilitation  Exercise  Diet | 4919  4919  1288  4919  4919  4919  4919  4919  4919  4919  4919 | 13  46  8  89 (50)  9  5  5  18  39 | 6  43  5  88 (53)  4  0  3  10  32 | 0-19  23-63  0-12  79-94 (37-70)  0-12  0-6  0-6  2-25  19-49 | 0-100  0-100  0-91  24-100 (0-100)  0-91  0-91  0-70  0-90  0-95 |
| 90 days follow up since admission | | | | | |
| Number of readmissions | 4919 | 1(1-2) | 1 | 1-1 | 1-3 |
| Readmissions with exacerbation in 90 days  Readmissions within 6 weeks | 4919 | 28  (60) | 26  1 | 18-33  1-1 | 0-54  1-3 |
| Total death within 90 days since admission | 5178 | 11.6 | 12 | 8-15 | 0-50 |

N: Number of cases that reported data. IQ: interquartile. FVC: forced vital capacity. FEV1: forced expired volume in the first second. SBP: systolic blood pressure. DBP: diastolic blood pressure. Hb: Haemoglobin
