# Supplementary material for: Clinical Audit of COPD Patients Requiring Hospital Admissions in Spain: AUDIPOC Study
Source: PLoS One. 2012 Jul 31;7(7):e42156. doi: 10.1371/journal.pone.0042156 (PMC3418048; doi:10.1371/journal.pone.0042156)
Supplement: Table S3 — Additional guidelines assessment. (DOCX) [file pone.0042156.s003.docx]

Table S3. Additional guidelines assessment

| Guidelines Statements | AUDIPOC results for patients grouped by hospital | | | | | | |
| --- | --- | --- | --- | --- | --- | --- | --- |
|  | Variable | | | Median | IRQ | Min-Max | |
| An exacerbation of COPD is characterised by a change in the patient’s baseline dyspnoea, cough, and/or sputum production or colour.  Increased breathlessness, the main symptom of an exacerbation, is often accompanied by wheezing and chest tightness, increased cough and sputum, change of colour and the tenacity of sputum and fever.  Increased breathlessness is a common feature of an exacerbation of COPD | Increased dyspnoea | | | 96 | 93-100 | 84-100 | |
|  | Increased sputum | | | 64 | 54-72 | 9-100 | |
|  | Increased purulence | | | 88 | 80-100 | 17-100 | |
|  | Change colour | | | 45 | 54-72 | 9-100 | |
|  | None of the symptoms | | | 0 | 0-4 | 0-13 | |
|  | Anthonisen Type I | | | 40 | 30-49 | 7-100 | |
|  | Anthonisen Type II | | | 26 | 19-32 | 0-50 | |
|  | Anthonisen Type III | | | 31 | 23-40 | 0-91 | |
| Exacerbations may also be accompanied by a number of nonspecific complaints, such as tachycardia and tachypnea, malaise, insomnia, sleepiness, fatigue, depression, and confusion. | Pedal oedema | | | 26 | 19-33 | 0-53 | |
|  | Tachycardia (> 100 lpm) | | | 31 | 19-33 | 0-53 | |
|  | Tachypnea (> 20 rpm) | | | 67 | 45-83 | 0-100 | |
| Diagnosis | Variable | | | Median | IRQ | | Min-Max |
| Patients may present for the first time with an exacerbation of COPD. In this situation, patients need assessing and their diagnosis confirmed. | First admission | | | 26 | 19-35 | 0-59 | |
|  | First admission with previous spirometry | | | 38 | 17-50 | 0-100 | |
|  | Spirometries at discharge in patients with first admission | | | 25 | 0-46 | 0-100 | |
| For patients that require hospitalization, measurement of arterial blood gases is important to assess the severity of an exacerbation.  Arterial blood gas tensions should be measured and the  inspired oxygen concentration should be recorded in all  patients with an exacerbation referred to hospital. | Cases with a blood gas  analysis in the emergency room | | | 95 | 88-100 | 33-100 | |
|  | Inspired oxygen concentration recorded in the ED | | | 93 | 73-100 | 0-100 | |
| Chest radiographs (posterior/anterior plus lateral) are useful in identifying alternative diagnoses that can mimic the symptoms of an exacerbation. A chest radiograph should be obtained for all patients with an exacerbation referred to hospital. | Chest radiographs in the first 24 hours | | | 1000 | 97-100 | 27-100 | |
| An ECG aids in the diagnosis of right heart hypertrophy, arrhythmias, and ischemic episodes. An ECG should be recorded (to exclude comorbidities) for all patients with an exacerbation referred to hospital. | ECG in the first 24 hours | | | 90 | 79-97 | 16-100 | |
| The whole blood count may identify polycythaemia (haematocrit > 55%) or bleeding.  A full blood count should be performed for all patients with an exacerbation referred to hospital. | Blood cell count in the ER | | | 95 | 88-100 | 33-100 | |
| Biochemical test abnormalities can be associated with an exacerbation and include electrolyte disturbance(s) (e.g., hyponatremia, hypokalaemia), poor glucose control, or metabolic acid-base disorder. Urea and electrolyte concentrations should be measured for all patients with an exacerbation referred to hospital. | Biochemical measurement in the ED | | | 95 | 88-100 | 33-100 | |
| If sputum is purulent, a sample should be sent for analysis for all patients with an exacerbation referred to hospital. | Cases with purulent sputum and a sputum microscopy in the first 24 hours | | | 17 | 0-42 | 0-100 | |
|  | Cases with purulent sputum and a sputum culture in the first 24 hours | | | 32 | 8-56 | 0-100 | |
| Oxygen therapy | Variable | | | Median | IRQ | Min-Max | |
| Oxygen therapy is the cornerstone of hospital treatment of COPD exacerbations and supplemental oxygen should be titrated to improve the patient’s hypoxemia | Cases receiving oxygen during admission | | | 95 | 95-100 | 53-100 | |
|  | Pulse-oxymetry while receiving oxygen-therapy | | | 98 | 86-100 | 0-100 | |
| Venturi masks (high-flow devices) offer more accurate delivery of controlled oxygen than do nasal prongs but are less likely to be tolerated by the patient. | Cases using venturi masks | | | 27 | 12-54 | 0-95 | |
|  | Cases using nasal prongs | | | 60 | 32-78 | 0-98 | |
| Bronchodilators |  | | |  |  |  | |
| Management of COPD exacerbations involves increasing the dose and/or frequency of existing short-acting bronchodilator therapy, preferably with a ß_2_ agonist. | | Cases on short-acting bronchodilators | 98 | | 94-100 | | 61-100 |
|  |  | Cases on short-acting ß_2_ agonists | 93 | | 85-97 | | 11-100 |
|  |  | Cases on ipratropium | 94 | | 88-100 | | 15-100 |
| If a prompt response to these drugs does not occur, the addition of an anticholinergic is recommended, even though evidence concerning the effectiveness of this combination is controversial. | | Cases on short-acting ß_2_ agonists and ipratropium | 88 | | 75-95 | | 7-100 |
| Despite its widespread clinical use, the role of methylxanthines in the treatment of exacerbations of COPD remains controversial.  Intravenous theophylline should only be used as an adjunct to the management of exacerbations of COPD if there is an inadequate response to nebulised bronchodilators. | | Cases receiving methylxanthines during admission | 9 | | 3-20 | | 0-75 |
| Antibiotics | | Variable | Median | | IRQ | | Min-Max |
| Antibiotics should be given to patients with three cardinal symptoms, with two cardinal symptoms if purulence of sputum is one of the two symptoms, and patients that require mechanical ventilation | | Cases on antibiotics with three cardinal symptoms | 98 | | 90-100 | | 50-100 |
|  |  | Cases on antibiotics with an increase in sputum purulence | 97 | | 91-100 | | 0-100 |
|  |  | Cases on ventilatory support receiving antibiotics | 100 | | 83-100 | | 0-100 |
| The risk factors for P. aeruginosa infection are recent hospitalization, frequent administration of antibiotics (4 courses in the last year), severe COPD exacerbations, and isolation of P. aeruginosa during a previous exacerbation or colonization during a stable period. | | Cases with another admission in the previous month | 34 | | 22-45 | | 0-75 |
|  |  | Cases with two or more admissions in the previous year | 19 | | 10-29 | | 0-63 |
|  |  | Cases with ventilatory support receiving antibiotics | 54 | | 39-66 | | 0-100 |
|  |  | Cases with any of these risk factors receiving quinolones during the admission | 100 | | 83-100 | | 0-100 |
| Steroids | | Variable | Median | | IRQ | | Min-Max |
| In the absence of significant contraindications oral corticosteroids should be used, in conjunction with other therapies, in all patients admitted to hospital with an exacerbation of COPD. | | Cases on oral or iv glucocorticosteroids | 94 | | 89-98 | | 50-100 |
| Discharge report | | Variable | Median | | IRQ | | Min-Max |
| Opportunities for prevention of future exacerbations should be reviewed before discharge, with particular attention to smoking cessation, current vaccination (influenza, pneumococcal vaccines), knowledge of current therapy including inhaler technique and how to recognize symptoms of exacerbations. | | Anti-tobacco instructions in active smokers | 43 | | 23-63 | | 0-100 |
|  |  | Influenza vaccination instructions | 0 | | 0-7 | | 0-100 |
|  |  | Pneumococcal vaccination instructions | 0 | | 0-3 | | 0-100 |
|  |  | Nutritional instructions | 37 | | 21-53 | | 0-100 |
|  |  | Inhaler technique instructions | 7 | | 0-19 | | 0-100 |
|  |  | Programmed visit after discharge | 95 | | 89-100 | | 30-100 |
| Patients should be re-established on their optimal maintenance bronchodilator therapy before discharge. | | Patients continuing on LABA at discharge | 92 | | 86-96 | | 50-100 |
|  |  | Patients continuing on tiotropium at discharge | 91 | | 82-100 | | 60-100 |
|  |  | Patients continuing with inhaled corticosteroids at discharge | 94 | | 89-100 | | 0-100 |
| Early outpatient pulmonary rehabilitation after hospitalization for a COPD exacerbation is safe and results in clinically significant improvements in exercise capacity and health status at 3 months | | Pulmonary rehabilitation at discharge | 3 | | 0-6 | | 0-70 |
|  |  | Instructions on discharge report about exercise | 10 | | 2-25 | | 0-90 |
